# Our Changing Climate

**Published:** 1881-07

**Authors:** 


### OUR CHANGING CLIMATE.

"Here let me say a word in favor of those vicissitudes of our climate which are too often made the subject of exclusive repining. If they annoy us occasionally by changes from hot to cold, from wet to dry, they give us one of the most beautiful climates in the world. They give us the brilliant sunshines of the south of Europe with the fresh verdure of the north. They float our summer sky with clouds of gorgeous tints or fleecy whiteness, and send down cooling showers to refresh the panting earth and keep it green. Our seasons are full of sublimity and beauty. Winter with us has none of its proverbial gloom. It may have its howling winds, and chilling frosts, and whirling snow-storms; but it has also its long intervals of cloudless sunshine, when the snow-clad earth gives redoubled brightness to the day—when at night the stars beam with intensest lustre, or the moon floods the whole landscape with her most limpid radiance. And then the joyous outbreak of our spring, bursting at once into leaf and blossom, redundant with vegetation, and vociferous with life, and the splendors of our summer—its morning voluptuousness and evening glory—its airy palaces of sun-lit clouds piled up in a deep azure sky; and its gusts of tempest of almost tropical grandeur, when the forked lightning and the bellowing thunder-volley from the battlements of heaven shake the sultry atmosphere! and the sublime melancholy of our autumn, magnificent in its decay, withering down the pomp and pride of a woodland country, yet reflecting back from its yellow forests the golden serenity of the sky. Surely we may say that in our climate, the heavens declare the glory of God; and the firmament showeth his handy work. Day unto day uttereth speech and night unto night showeth knowledge."

